# Insights into the Molecular Architecture of a Peptide Nanotube Using FTIR and Solid-State NMR Spectroscopic Measurements on an Aligned Sample**

**DOI:** 10.1002/anie.201301960

**Published:** 2013-08-16

**Authors:** David A Middleton, Jillian Madine, Valeria Castelletto, Ian W Hamley

**Affiliations:** Institute of Integrative Biology, University of Liverpool Crown Street, Liverpool L69 7ZB (UK); Department of Chemistry, University of Reading Whiteknights, Reading RG6 6AD (UK)

**Keywords:** IR spectroscopy, nanotubes, NMR spectroscopy, peptides

The self-assembly of proteins and peptides into β-sheet-rich amyloid fibers is a process that has gained notoriety because of its association with human diseases and disorders. Spontaneous self-assembly of peptides into nonfibrillar supramolecular structures can also provide a versatile and convenient mechanism for the bottom-up design of biocompatible materials with functional properties favoring a wide range of practical applications.^1^ One subset of these fascinating and potentially useful nanoscale constructions are the peptide nanotubes, elongated cylindrical structures with a hollow center bounded by a thin wall of peptide molecules.^2^ A formidable challenge in optimizing and harnessing the properties of nanotube assemblies is to gain atomistic insight into their architecture, and to elucidate precisely how the tubular morphology is constructed from the peptide building blocks. Some of these fine details have been elucidated recently with the use of magic-angle-spinning (MAS) solid-state NMR (SSNMR) spectroscopy.^3^ MAS SSNMR measurements of chemical shifts and through-space interatomic distances provide constraints on peptide conformation (e.g., β-strands and turns) and quaternary packing. We describe here a new application of a straightforward SSNMR technique which, when combined with FTIR spectroscopy, reports quantitatively on the orientation of the peptide molecules within the nanotube structure, thereby providing an additional structural constraint not accessible to MAS SSNMR.

For many years the SSNMR analysis of lipid bilayers uniaxially aligned on glass cover slides has provided a useful approach to obtain structural and topological information on membrane proteins, by exploiting the orientation dependence of NMR interactions under nonspinning (static) conditions.^4^ The principal axes of nuclear spin interaction tensors (e.g., ^15^N chemical shielding, ^1^H–^15^N dipolar, or ^2^H quadrupolar) are often known relative to a local molecular reference frame and so the resonance frequencies measured from a membrane orientated at a defined angle within the magnetic field provide angular restraints on protein domains within the lipid matrix. We reasoned that the rigid-body structure of a peptide nanotube could also adopt a unique orientation when supported on glass slides and thus it would be possible to analyze the observed line shape to obtain angular values defining the orientation of the peptide molecules within the nanotube framework. To test this hypothesis we selected the peptide H_2_N-Ala-Ala-Ala-Ala-Ala-Ala-Lys-COOH (A_6_K), which assembles spontaneously and rapidly into nanotubes approximately 20–25 nm in diameter (Figure 1 a) constructed from monolayers of hydrogen-bonded peptide β-strands with a 4.7 Å strand spacing.^5^ A_6_K is one of several related amphiphilic peptide detergents that may provide a useful matrix for stabilizing membrane proteins in the solid state.^6^ Our earlier rotational resonance MAS SSNMR measurements on unoriented A_6_K nanotubes labeled with [1-^13^C]Ala at residue 2 (site C_A_) and [2-^13^C]Ala at residue 6 (site C_B_) were consistent with an antiparallel arrangement of β-strands (Figure S1 in the Supporting Information). We therefore aimed to extend the scope of SSNMR to obtain quantitative information on the orientation of A_6_K molecules in the nanotube framework.

**Figure 1:**
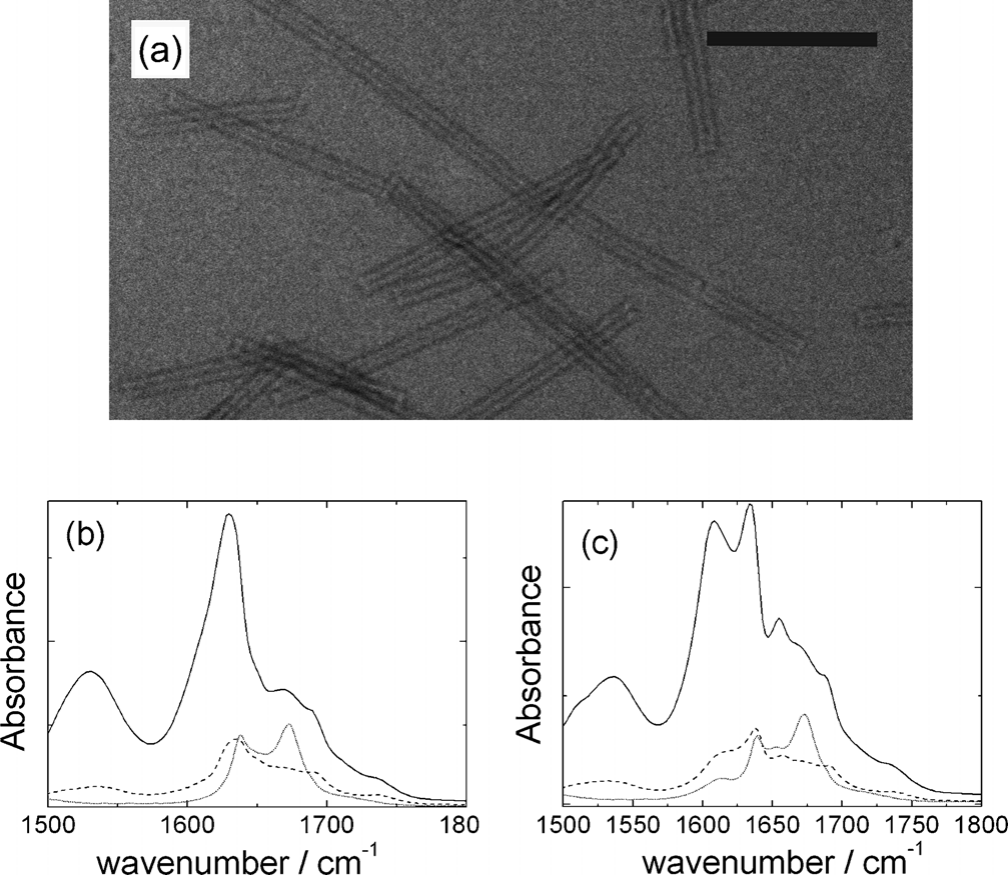
a) Transmission electron microscopy (TEM) image of A_6_K nanotubes in water; scale bar is 200 nm. b) FTIR spectra for unlabeled A_6_K. c) FTIR spectra for [^13^C_2_]A_6_K. The solid lines are ATR spectra for dried films, the dashed lines are transmission spectra for dried films, and the dotted lines are transmission spectra for solutions in D_2_O.

We began by performing FTIR spectroscopy on A_6_K to obtain qualitative information on the β-strand alignment using a standard technique. In particular, comparison of spectra in attenuated total reflection (ATR) mode versus those recorded in transmission mode gives information on the alignment of particular vibrational modes.^7^ Figure 1 b shows spectra in the amide II′ and amide I′ regions. The ATR spectrum for A_6_K shows a peak in the amide II′ region at 1530 cm^−1^ and in the amide I′ region peaks are observed at 1630, 1672, and 1690 cm^−1^. The peak at 1672 cm^−1^ is due to bound trifluoroacetate (TFA) counterions.^8^ The strong peak at 1630 cm^−1^ along with the weak peak at 1690 cm^−1^ are typical features of antiparallel β-sheet structures.^9^ Although the FTIR spectra are not normalized, the relative intensity of features within spectra enables comparison between ATR and transmission mode. The latter spectra (for dried film and solution) show strong attenuation of the peak near 1630 cm^−1^ and more particularly of the amide II′ peak at 1530 cm^−1^. The former peak is associated with the C–O stretch deformation and the latter is primarily due to N–H out-of-plane bending, perpendicular to the amide I′ mode.7b Enhanced intensity in grazing incidence mode indicates C–O bonds perpendicular to the IR beam, that is, strands lying approximately perpendicular to the tube axis.^7^ The FTIR data for the unlabeled peptide are therefore consistent with antiparallel β-strands aligned perpendicular to the nanotube axis, although the precise tilt angle of the C–O bond cannot be confirmed. The relative intensities of FTIR peaks for [^13^C_2_]A_6_K shown in Figure 1 c show features similar to those of the unlabeled peptide and support the same model of strand alignment. As previously observed for ^13^C-labeled alanine-based peptide FTIR spectra,^10^ a red-shifted β-sheet peak is observed at 1608 cm^−1^ although this occurs along with the original 1630 cm^−1^ peak in the ATR spectrum in Figure 1 c. Interestingly, the peak from the labeled alanines is more intense than that from the unlabeled residues—this has been ascribed^10^ to interstrand coupling between ^13^C and ^12^C carbonyl groups consistent with out-of-register strands.

Next we assessed whether the nanotubes could adopt a preferred alignment on cover slides suitable for SSNMR analysis and give rise to line shapes containing orientational information. For this purpose the peptide ([^15^N]A_6_K was prepared with [^15^N]Ala incorporated at residue 3 in the sequence and the nanotube gel (17 % w/v in water) was deposited by pipette onto 25 glass cover slides which were then individually allowed to dry at 25 °C in a dehydration chamber over 3 days, and then equilibrated in a rehydration chamber for a further 3 days. The slides were stacked together and oriented at 90° relative to the applied magnetic field in a fixed-angle flat-coil NMR probehead for analysis. The ^15^N NMR spectrum obtained from the sample (Figure 2 a) differs somewhat from the powder pattern line shape for randomly oriented nanotubes in the gel (Figure 2 b), and shows broad anisotropic features that are consistent with a nonrandom distribution of ^15^N chemical shielding tensor orientations. A ^2^H NMR spectrum was obtained for nanotubes of A_6_K labeled with [3-^2^H_3_]Ala at position 3 ([^2^H_3_]A_6_K). The ^2^H quadrupolar line shape (Figure 2 c) differs markedly from the powder spectrum obtained from an unoriented nanotube gel (Figure 2 d) and consists of a single Pake doublet consistent with a restricted distribution of axially symmetric quadrupolar tensors. Taken together, the two NMR measurements thus confirm that the nanotubes adopt a preferred orientation.

**Figure 2:**
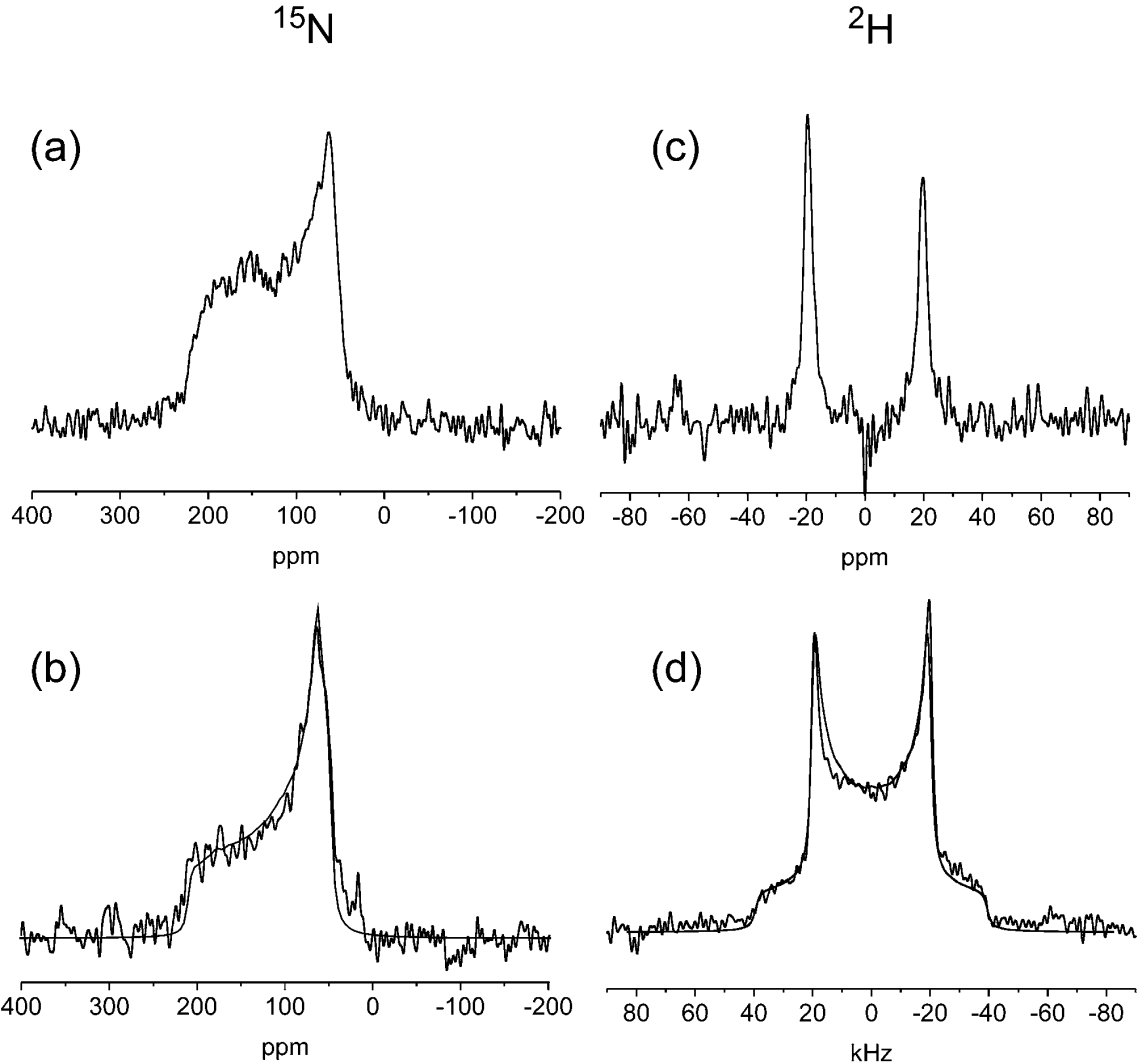
Static NMR spectra of A_6_K nanotubes. a) ^15^N spectrum of [^15^N]A_6_K deposited on glass cover slides oriented at 90° to the magnetic field. b) ^15^N spectrum of an unoriented hydrated gel of [^15^N]A_6_K; the solid line is a simulated powder spectrum for chemical shift tensor elements σ_11_=62 ppm, σ_22_=72 ppm, and σ_33_=220 ppm. c) ^2^H spectrum of [^2^H_3_]A_6_K on glass slides. d) ^2^H spectrum of an unoriented gel of [^2^H_3_]A_6_K; the solid line is a simulated powder spectrum for an axially symmetric quadrupolar interaction and a quadrupole splitting of 37.5 kHz.

The NMR spectra of the aligned samples were compared with numerically simulated line shapes calculated from structural models of the nanotubes with different peptide orientations. For the simulation procedure it was assumed that the nanotubes align with their long (*Z*) axes perpendicular to the surface normal of the glass slides and thus also perpendicular to the applied magnetic field B_0_ (Figure 3 a). All nanotube models were 20 nm in diameter and consisted of antiparallel hydrogen-bonded β-strands with a 4.7 Å spacing in the hydrogen-bonding direction.^5^ An intersheet spacing of 10 Å orthogonal to the hydrogen-bonding direction was assumed based on X-ray fiber diffraction data for other peptide nanotubes.^3^ The spacings do not affect the line shape of the simulated spectra. A series of nanotube models was generated with the β-strands perpendicular to the nanotube axis *Z* (i.e., the nanotube wall thickness being the length of one β-sheet monolayer, 25 Å) and the interstrand hydrogen bonds oriented at 0°≤*θ*≤90° relative to *Z*. Hence when *θ*=90° the peptides form radially organized β-sheets running perpendicular to the nanotube axis (Figure 3 a) and when *θ*=0° the β-sheet layers run parallel with the nanotube axis (Figure 3 b). When *θ* decreases from 90° the off-axis N–H tilt allows the peptides to adopt a helical ensemble structure (Figure 3 c). Analysis of the NMR line shapes in Figure 2 can in principle determine the precise N–H tilt angle, providing additional quantitative information that is not available from the FTIR measurements. For each tilt angle, further models were generated in which peptides were rotated slightly so that *θ* varied randomly from the mean value by angles between 0° and ±*n*. The distribution angle *n* was varied between 0 and |25°| to reflect different levels of disorder in the nanotube assembly. The coordinates for each model were taken to calculate ^15^N and ^2^H spectra, using published chemical shielding and quadrupolar tensor orientations. The closest fits between experimental and simulated data for ^15^N and ^2^H spectra concur for models in which *θ* takes values in the range 65–70° and *n* lies in the range 10–15° (Figure 4 and Figures S2 and S3 in the Supporting Information). Poorer fits were observed for models with exact parallel or perpendicular orientations of N–H bonds relative to the nanotube axis (i.e., models (a) and (b) in Figure 3), and the fits were considerably worse for models in which the peptide β-strands are parallel with the nanotube axis (Figures S4–S8 in the Supporting Information). The N–H tilt angle of 70° permits a helical arrangement of the A_6_K peptides as illustrated in Figures 3 c and 4 b. The FTIR spectra are also consistent with such a model but do not provide de novo quantitative information on *θ* or *n*. These findings suggest that the observed XRD pattern from aligned A_6_K nanotubes5b should be re-interpreted;5c in particular the 5.5 Å peaks oriented at 52° with respect to the nanotube axis are consistent with the helical arrangement determined here by NMR spectroscopy. The 5.5 Å peak arises from the alanine stacking distance.

**Figure 3:**
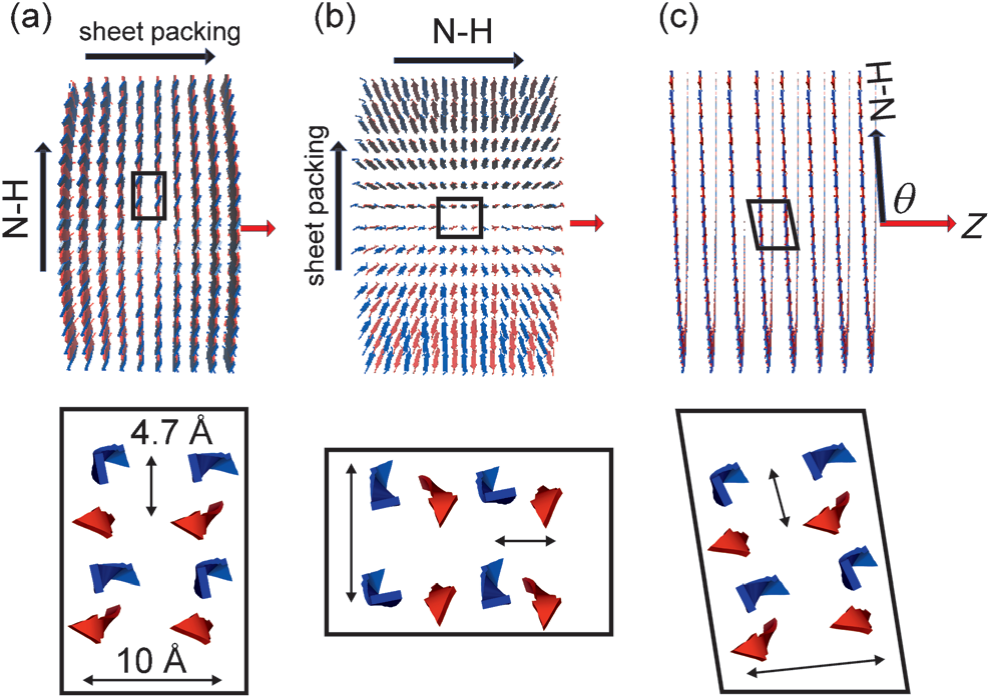
Sections of the nanotube models used for the NMR line shape simulations. The nanotube long axis *Z* (red arrow) is oriented perpendicular to the applied field B_0_. a) Hydrogen-bonding (N–H) axis perpendicular to *Z*. b) Hydrogen-bonding axis parallel with *Z*. c) A generalized model, for which the N–H bond is oriented at angle *θ* relative to *Z*. The expanded regions (bottom) show the orientations of antiparallel β-strands (denoted by alternating red–blue arrows) in which the repeat distance along the hydrogen bonding axis is 4.7 Å and the distance between sheet layers is 10 Å. The models arbitrarily assume a parallel arrangement of the β-sheet layers.

**Figure 4:**
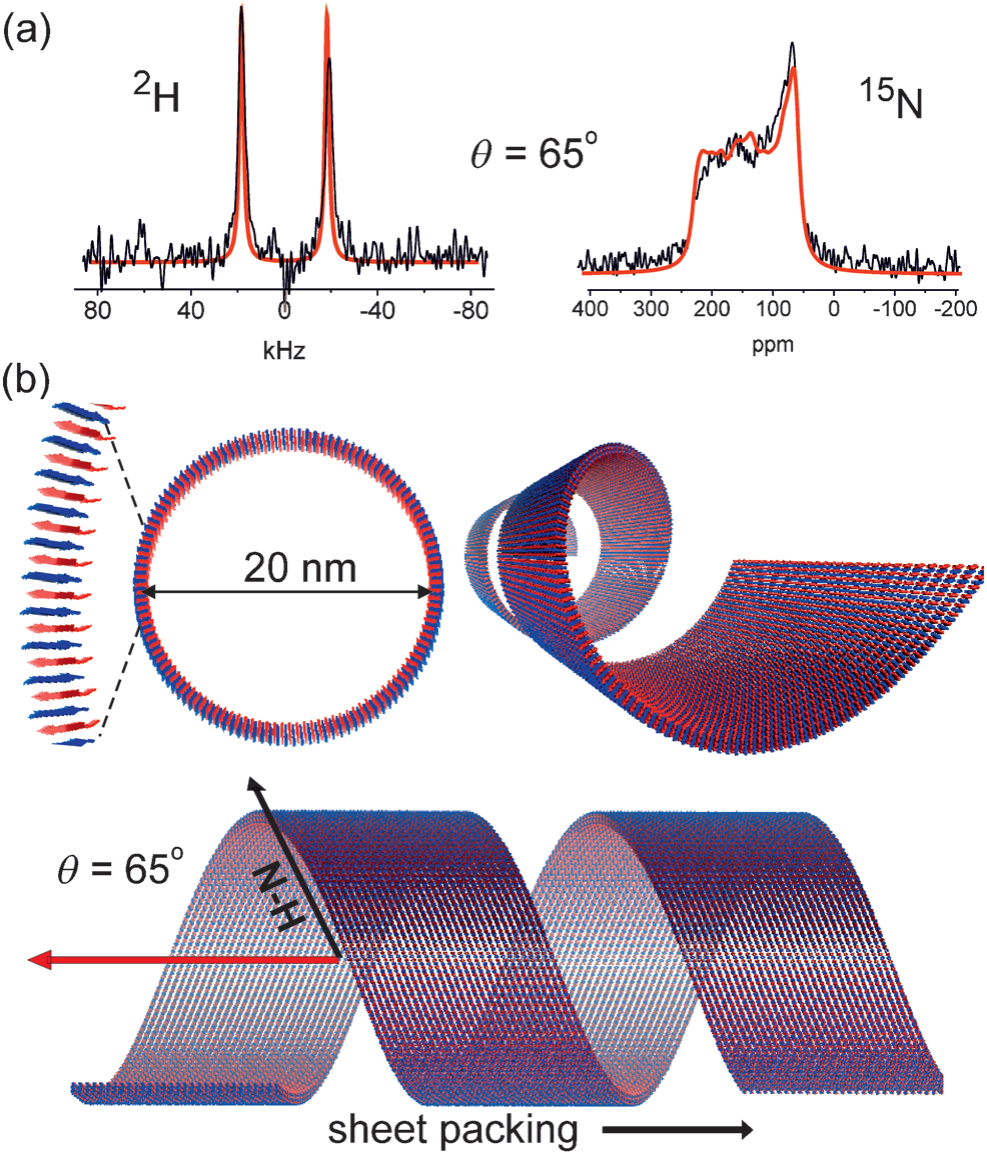
Analysis and interpretation of the NMR spectra a) Experimental spectra (black) superimposed with simulated spectra for *θ*=65° and *n*=15°. b) Three perspectives of the nanotube model integrating all known information.

In summary we report a powerful new procedure, combining SSNMR and FTIR spectroscopy, to determine the tilt angle of the peptide hydrogen-bonding axis within a nanotube framework. Ambiguities in the measurement of the N–H tilt angle (e.g., arising from molecular disorder) could in future be minimized by using variable-angle coil probes to alter the orientation of the glass slides in the magnetic field. By demonstrating the feasibility of the procedure with the paradigm of A_6_K we pave the way to further structural investigations of other peptide nanostructures as well as the orientations of guest molecules associated with them.

## Experimental Section

All peptides (95 % pure) were purchased from Peptide Protein Research (Fareham, UK). The morphology of the A_6_K aggregates was analyzed by TEM using negative staining with 4 % uranyl acetate. Peptide suspensions (10 μL) were loaded onto carbon-coated copper grids and visualized on a Tecnai 10 electron microscope at 100 kV. NMR experiments were performed using a Bruker Avance 400 spectrometer operating at a magnetic field of 9.3 Tesla. Peptide aggregates were sedimented from bulk solution by centrifugation and deposited onto 9×22 mm glass cover slides (Marienfeld, Germany), and allowed to dehydrate and rehydrate. Stacked slides were wrapped in a protective film and inserted into a Bruker double-resonance flat-coil probe with coil dimensions of 9×9×3 mm. NMR measurements were performed at −10 °C to reduce the heat generated by proton decoupling. The ^15^N spectra of oriented and unoriented samples were obtained with proton decoupling during acquisition at a field of 83 kHz and a recycle delay of 3 s. The ^2^H spectra of oriented and unoriented samples were obtained using the quadrupole echo sequence with interpulse delays of 30 μs and recycle delay of 2 s. Further details of the NMR measurements and details of the FTIR measurements are given in the Supporting Information.

## Supporting Information

As a service to our authors and readers, this journal provides supporting information supplied by the authors. Such materials are peer reviewed and may be re-organized for online delivery, but are not copy-edited or typeset. Technical support issues arising from supporting information (other than missing files) should be addressed to the authors.

**miscellaneous_information**
