# Supplementary material for: Insights into the Molecular Architecture of a Peptide Nanotube Using FTIR and Solid-State NMR Spectroscopic Measurements on an Aligned Sample
Source: Angew Chem Int Ed Engl. 2013 Aug 16;52(40):10537–40. doi: 10.1002/anie.201301960 (PMC4672711; doi:10.1002/anie.201301960)
Supplement: Supplementary file 1 [file anie0052-10537-sd1.pdf]

Angewandte Chemie
Eine Zeitschrift der Gesellschaft Deutscher Chemiker

Supporting Information

© Wiley-VCH 2013

69451 Weinheim, Germany

Insights into the Molecular Architecture of a Peptide Nanotube Using FTIR and Solid-State NMR Spectroscopic Measurements on an Aligned Sample**

David A. Middleton, Jillian Madine, Valeria Castelletto, and Ian W. Hamley*

anie_201301960_sm_miscellaneous_information.pdf

Supporting Information

FTIR measurements

FTIR spectra were recorded using a Nexus-FTIR spectrometer equipped with a DTGS detector and a multiple reflection ATR system. Transmission FTIR measurements were performed using a CaF_2 plate, while grazing angle FTIR experiments were done using a ZnSe plate. For both transmission and grazing angle configurations, small aliquots of solutions (17 wt % A_6K or 17 wt% $A^*A^*_5K$) were deposited on the corresponding plate and allowed to dry, providing a solid film of dry peptide. Transmission FT-IR experiments were also performed on 17 wt % A_6K or 17 wt% $A^*A^*_5K$ solutions in D_2O, placing the samples between two CaF_2 windows (0.006 mm spacer). Spectra were scanned 64 times and 128 times for transmission and grazing angle geometries respectively.

Solid-state NMR measurements. Rotational resonance SSNMR measurements were performed on the peptide [$^{13}C_2$]A6K, which incorporates [1-^{13}C]Ala at residue 2 (site C_A) and [2-^{13}C]Ala at residue 6 (site C_B). A 17 % w/v solution of the peptide in water was incubated at 25°C and self-assembled into nanotubes after 48 h, as confirmed by transmission electron microscopy (Figure 1a). The hydrated gel was concentrated by centrifugation and a rotational resonance SSNMR method was used to obtain the inter-strand C_A-C_B distance restraint by detection of ^{13}C-^{13}C dipolar coupling. All experiments were performed using a Bruker Avance 400 spectrometer operating at a magnetic field of 9.3 Tesla. All experiments utilized an initial 4.0-μs ^{1}H 90° excitation pulse length, 1-ms Hartmann-Hahn contact time at a matched ^{1}H field of 65 kHz, TPPM proton decoupling at a field of 85 kHz during signal acquisition and a 2-s recycle delay. RR experiments [2,3] were carried out by adjusting the sample spinning rate (ω_R) to the exact difference between the resonance frequencies ($\Delta\Omega$) of C_A and C_B (n = 1 rotational resonance) or to half the frequency difference (n = 2 RR). After cross-polarization, ^{13}C longitudinal difference polarization was created with a nonselective 4-μs $\pi/2$ pulse followed by a train of 18 DANTE pulses, representing an overall π pulse of 30 μs, to invert the ^{13}C spin polarisation for Cα selectively. After a mixing period the ^{13}C magnetization was returned to the transverse plane by a second nonselective $\pi/2$ pulse before digitization of the free-induction decay. A series of experiments using mixing periods of up to 34 ms was performed to measure the time dependence of difference polarization.

Curves representing exchange of Zeeman order were obtained from the difference in intensities of the Ala2 methyl carbon and Val5 carbonyl carbon peaks.

Simulation of RR exchange curves. Interatomic distances were determined by comparison of the data with numerically simulated curves. Curves were simulated for dipolar coupling constants d_{CC} corresponding to fixed pairs of ^{13}C-^{13}C distances derived from molecular models of feasible β-strand registrations. The ^{13}C-^{13}C interatomic distance r_{CC} is related to d_{CC} according to the equation:

$$d_{CC} = -(\frac{\mu_0}{4\pi})\frac{\gamma_I \gamma_S \hbar}{r_{CC}^3} \qquad [3]$$

The zero quantum relaxation time (T_2^{ZQ}) also affects the shape of the curve. T_2^{ZQ} is generally not known, but can be estimated from the reciprocal sum of the NMR line widths. A series of curves was calculated by varying distance r between 3.0 Å and 7.0 Å. T_2^{ZQ} was varied between 1 ms and 5 ms, a conservatively broad range of values, and all other parameters were constant.

Nanotube modelling

Nanotube structural models were generated using a home-written C program (available free of charge by request to the authors). The atomic coordinates of a single A_6K molecule were expressed in a reference frame with the z axis coincident with the principal axis of inertia (i.e., the long axis of the peptide β-strand). The peptide coordinates were replicated radially using a series of translations and rotations to form a single molecule-thick, circular cross-section of the nanotube composed of antiparallel β-strands, as summarised in Figure 3 (b-d) of the main text. The nanotube was then elongated by propagating several such layers along the nanotube axis. The nanotube diameter was set to approximately 400 nm as determined by the electron microscopy, with molecules separated by 4.7 Å in the hydrogen bonding direction and 10 Å in the orthogonal packing direction, as determined by X-ray fibre diffraction. Peptides were rotated about their long axes to vary the mean peptide tilt angle θ of the N-H bonds from 0° (parallel to the nanotube axis) to 90° (perpendicular to the nanotube axis). The peptide was also set to be either parallel or perpendicular to the nanotube axis. Each peptide orientation was varied randomly to produce for each molecular

position an ensemble of N-H bond angles in a normal distribution with mean θ and upper and lower limits of $\pm n$, the distribution angle. The intrinsic disorder within the nanotubes was thus increased by varying n from zero to 25°. Finally, the nanotube coordinates were rotated as a rigid body and expressed in a laboratory reference frame as being either parallel or perpendicular to the magnetic field. Hence complete control could be exercised over the peptide molecular orientation within the nanotube, and the orientation of the nanotube in the magnetic field.

Table 1 summarises the input variables for all the nanotube models prepared. Model coordinates were saved in pdb file format.

Table 1. Summary of the nanotube models produced and analysed. Simulations for each model set are shown in Figures S2-S4.

**Model set**	**Number of models**	**Variables**	**Peptide orientation[b]**	**Nanotube orientation[c]**
PerPer[a]	60	θ (0-90° in 10° increments)<br>*n* (0-25° in 5° increments)	Perpendicular	Perpendicular
PerPara	60	θ (0-90° in 10° increments)<br>*n* (0-25° in 5° increments)	Perpendicular	Parallel
ParaPer	60	θ (0-90° in 10° increments)<br>*n* (0-25° in 5° increments)	Parallel	Perpendicular
ParaPara	60	θ (0-90° in 10° increments)<br>*n* (0-25° in 5° increments)	Parallel	Parallel

[a]The model set used for the NMR line shape analysis described in the main text.

[b]Relative to the nanotube axis

[c]Relative to the magnetic field. Perpendicular assumes the long axis is coincident with the surface plane of the cover slides.

Simulation of NMR spectra

^{15}N spectra: The main elements σ_{11}, σ_{22} and σ_{33} of the ^{15}N chemical shift interaction tensor were measured from a static, unoriented sample of [^{13}C]A_6K nanotubes. The measured values of 62 ppm, 72 ppm and 220 ppm agreed closely with published values [1]. Simulated ^{15}N line shapes for the different nanotube models were computed using a custom-written C program which performed calculations on the atomic coordinates extracted from the model pdb files. In the computation it was first necessary to calculate the ^{15}N tensor orientation relative to the direction of the applied magnetic field for Ala3 of each A6K molecule in the model. It was assumed for this purpose that the main tensor elements σ_{11} and σ_{33} are coincident with the plane of the peptide bond, with σ_{33} making an angle of 10° with the N-H bond. The Euler angles ϕ and ψ, which describe the orientation of the principal elements of the ^{15}N chemical shift tensor relative to the magnetic field, could then be calculated from the atomic coordinates of the amide N and H of Ala4 and carbonyl carbon C’ of Ala3. These angles vary according to the peptide molecular orientations within the nanotube and the

orientation of the nanotubes on the glass cover slides (which are oriented at 90° in the magnetic field). The measured ^{15}N chemical shift σ_{zz} for each A6K molecule was calculated as:

$$\sigma_{zz} = \sigma_{11} sin^2\psi cos^2\phi + \sigma_{22} sin^2\psi sin^2\phi + \sigma_{33} cos^2\psi \qquad [1]$$

In total, chemical shifts were calculated for an ensemble of 2400 molecules in each nanotube model.

^{2}H spectra: A similar procedure was used to simulate ^2H line shapes. In this case an axially symmetric quadrupolar interaction was assumed for a rapidly rotating CD_3 group, with the principal axis z coincident with the Cα-Cβ bond orientation. Angle ψ defines the orientation of the quadrupolar tensor in the magnetic field, and the measured frequency for each orientation is given by:

$$\Delta\upsilon_Q = \frac{3e^2qQ}{4h}(3cos^2\psi - 1) \qquad [2]$$

The term e^2qQ/h is the static quadrupolar coupling constant. A quadupolar splitting of 37.5 kHz was measured from an unoriented nanotube sample (Figure 2 of the main text).

Supplementary Results

β-strand alignment. Rotational resonance SSNMR was used to determine whether the β-strands were hydrogen-bonded in a parallel or antiparallel configuration. For this purpose the peptide [$^{13}C_2$]A6K was prepared, which incorporates [1-^{13}C]Ala at residue 2 (site C_A) and [2-^{13}C]Ala at residue 6 (site C_B). A 17 % w/v solution of the peptide in water was incubated at 25°C and self-assembled into nanotubes after 48 h, as confirmed by transmission electron microscopy (Figure S1a). The hydrated gel was concentrated by centrifugation and rotational resonance SSNMR was used to obtain the inter-strand C_A-C_B distance restraint by detection of ^{13}C-^{13}C dipolar coupling.

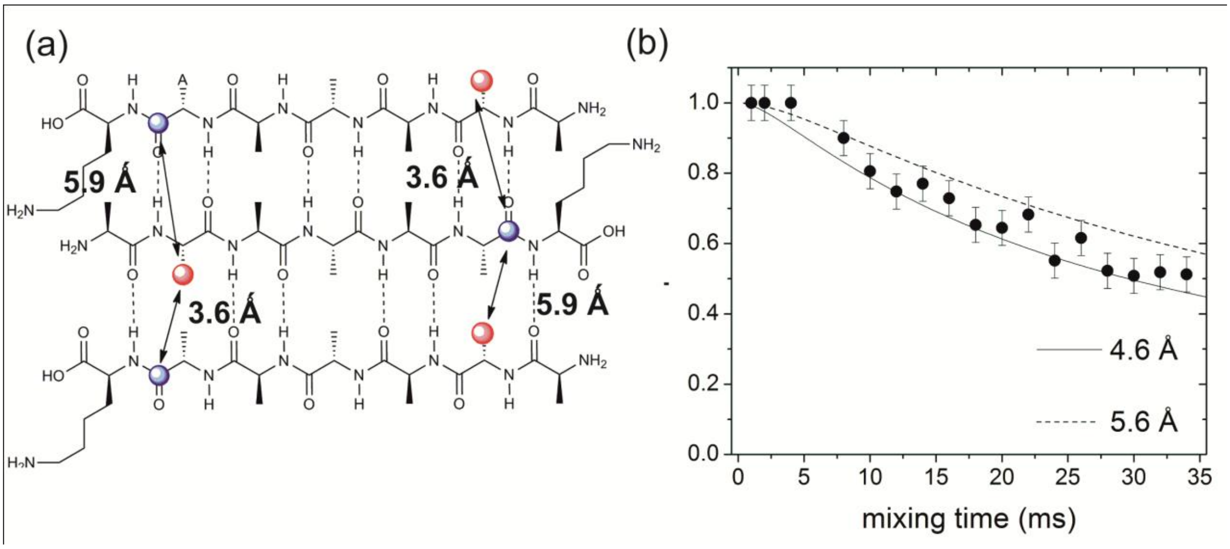

Figire S1. Determination of the A6K β-strand alignment using SSNMR. (a) Structure of [$^{13}C_2$]A6K in an antiparallel configuration, showing the intermolecular distances between C_A (red spheres) and C_B (blue spheres). (b) Rotational resonance (RR) SSNMR on A6K. Magnetization exchange is shown at n = 1 (filled circles) rotational resonance. Error bars represent the level of the noise. Solid and dashed lines are numerically simulated curves representing the C_A-C_B distances within the upper and lower limits of the errors.

The intermolecular C_A-C_B distance is strongly dependent on the orientation of the β-strands, ~ 5 Å for antiparallel β-strands (Figure S1b). The measured difference polarization at n = 1 rotational resonance is seen to decay with time (Figure 1b), and comparison of the data with numerical simulations reveals that the C_A-C_B through-space distance is 5.1 Å ± 0.5 Å, thereby confirming that the nanotubes are constructed from antiparallel β-strands with adjacent

peptides aligned with Ala1 in–register with Lys6. A parallel β-strand alignment would result in a C_A-C_B through-space distance of ~16 Å, which would not give rise to a detectable response in the rotational resonance measurement.

Simulations

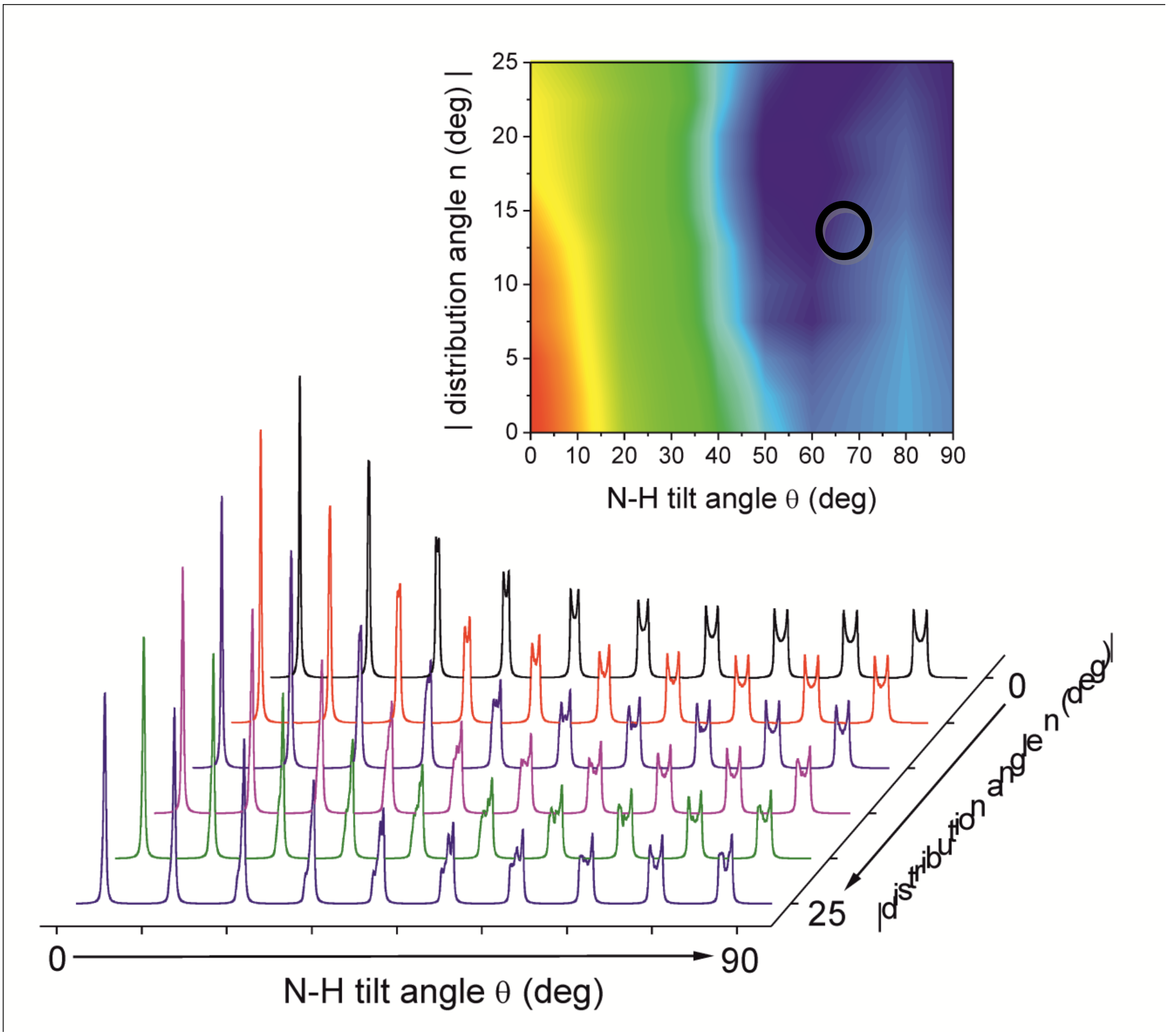

Figure S2. Simulated ^{15}N lineshapes calculated for the 60 models with the A6K β-strand axis perpendicular to the nanotube axis and the nanotube axis perpendicular to the magnetic field (model set *PerpPerp*, which forms the basis for the analysis in the main text). The inset shows the chi-square map obtained by comparison of the experimental ^{15}N spectrum with each of the simulated line shapes. The darkest blue regions correspond to chi-square values of $< 8 \times 10^4$ and the red region to chi-square values of $> 7.3 \times 10^6$. The same contour levels were applied to the maps in Figures S2 and S4-S8. The circled region corresponds to the simulated ^{15}N spectrum shown in Figure 4a of the main text.

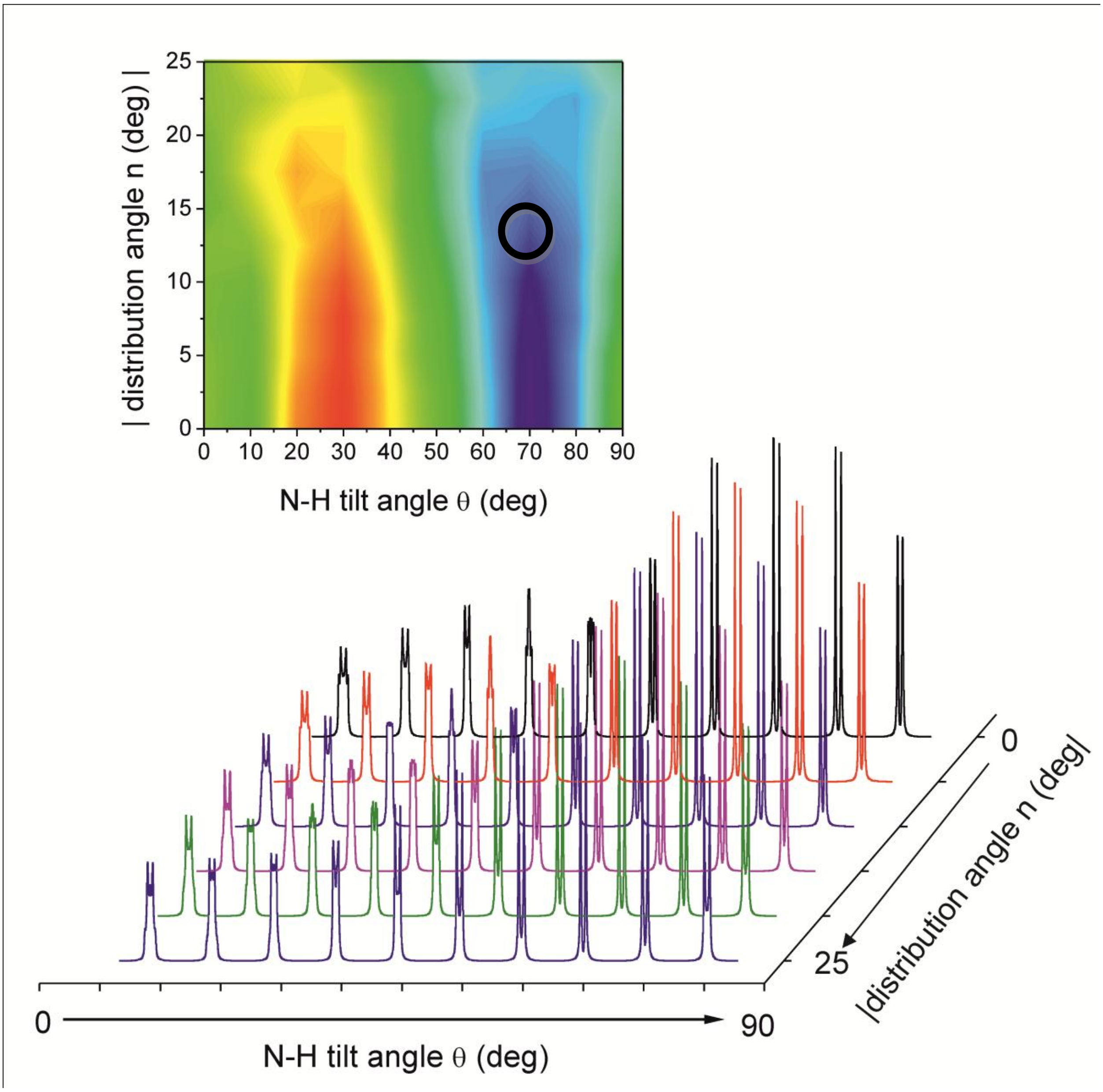

Figure S3. Simulated ^{2}H lineshapes calculated for the 60 models with the A6K β-strand axis perpendicular to the nanotube axis and the nanotube axis perpendicular to the magnetic field (model set *PerpPerp*, which forms the basis for the analysis in the main text). The inset shows the chi-square map obtained by comparison of the experimental ^{2}H spectrum with each of the simulated line shapes. The circled region corresponds to the simulated ^{2}H spectrum shown in Figure 4a of the main text.

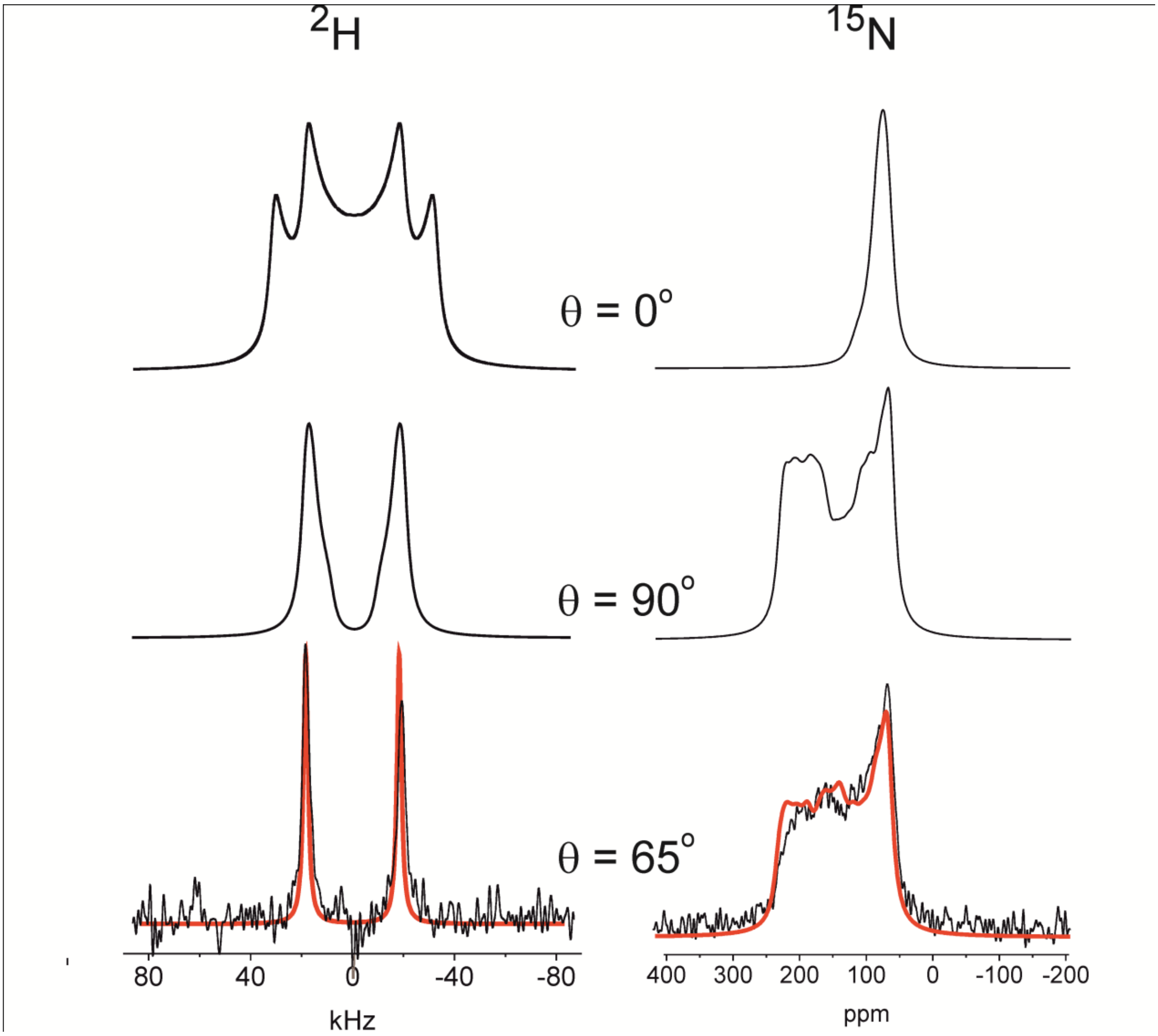

Figure S4. Simulated lineshapes for θ, *n* = 0°, 15° (corresponding to the model shown in Figure 3a of the main text), θ, *n* = 90°, 15° (corresponding to the model shown in Figure 3b) and θ, *n* = 65°, 15° superimposed with the experimental spectra.

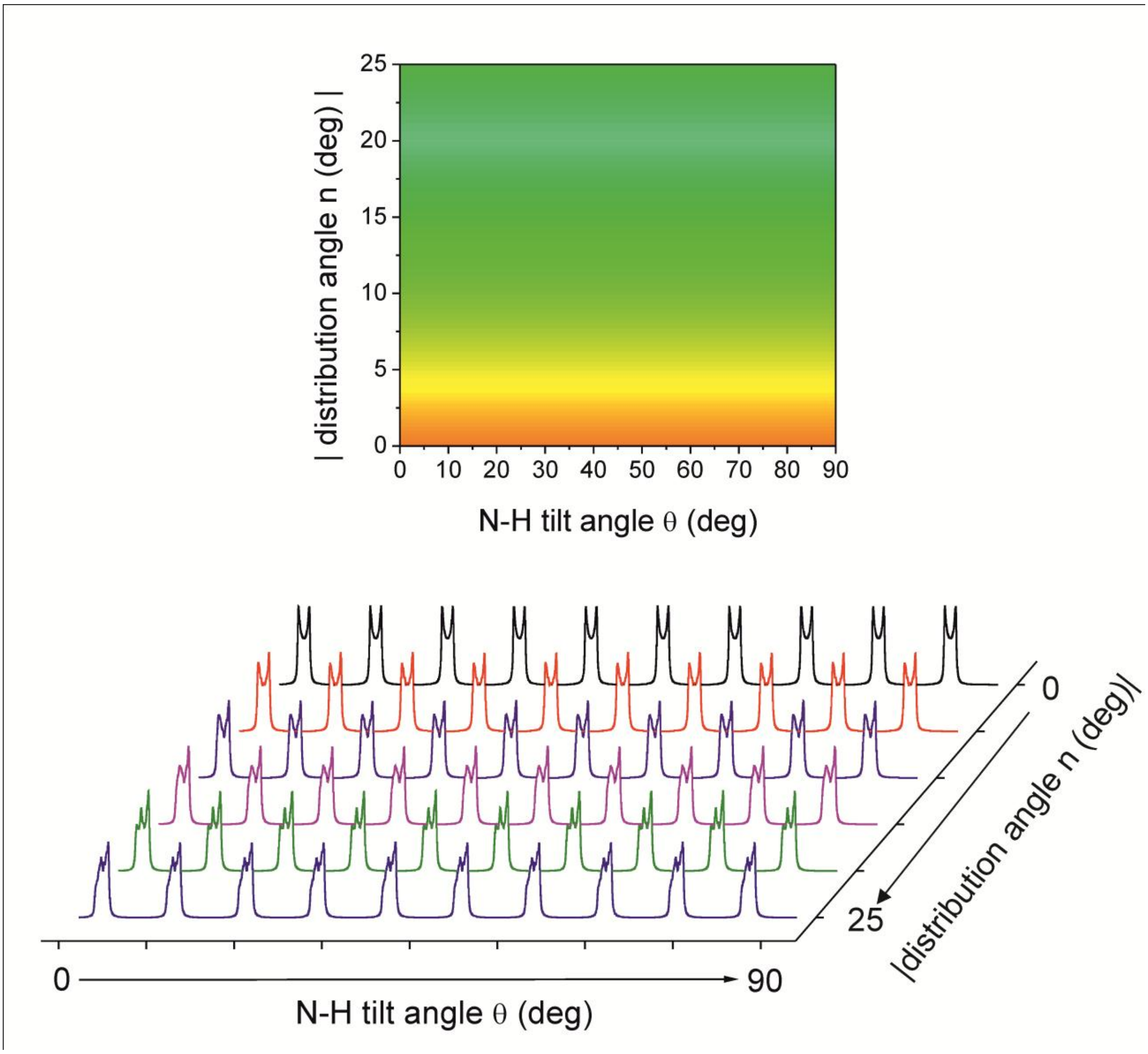

Figure S5. Simulated ^{15}N lineshapes calculated for the 60 models with the A6K β-strand axis parallel to the nanotube axis and the nanotube axis perpendicular to the magnetic field (model set *ParaPerp*). The inset shows the chi-square map obtained by comparison of the experimental ^{15}N spectrum with each of the simulated line shapes.

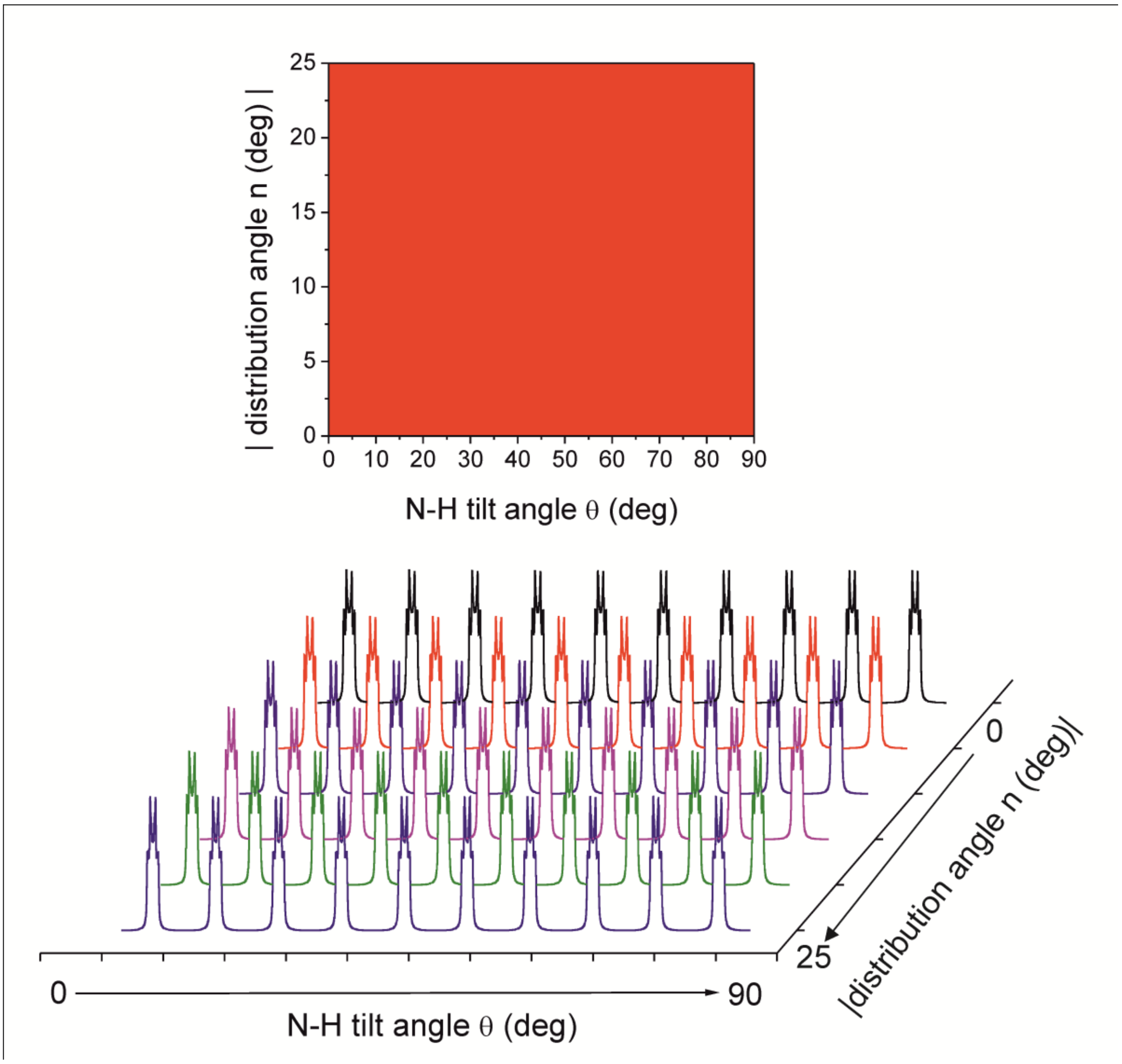

Figure S6. Simulated ^{2}H lineshapes calculated for the 60 models with the A6K β-strand axis perpendicular to the nanotube axis and the nanotube axis perpendicular to the magnetic field (model set *ParaPerp*). The inset shows the chi-square map obtained by comparison of the experimental ^{2}H spectrum with each of the simulated line shapes. The uniform red colour indicates that all chi-square values were greater than 7.3×10^{6}.

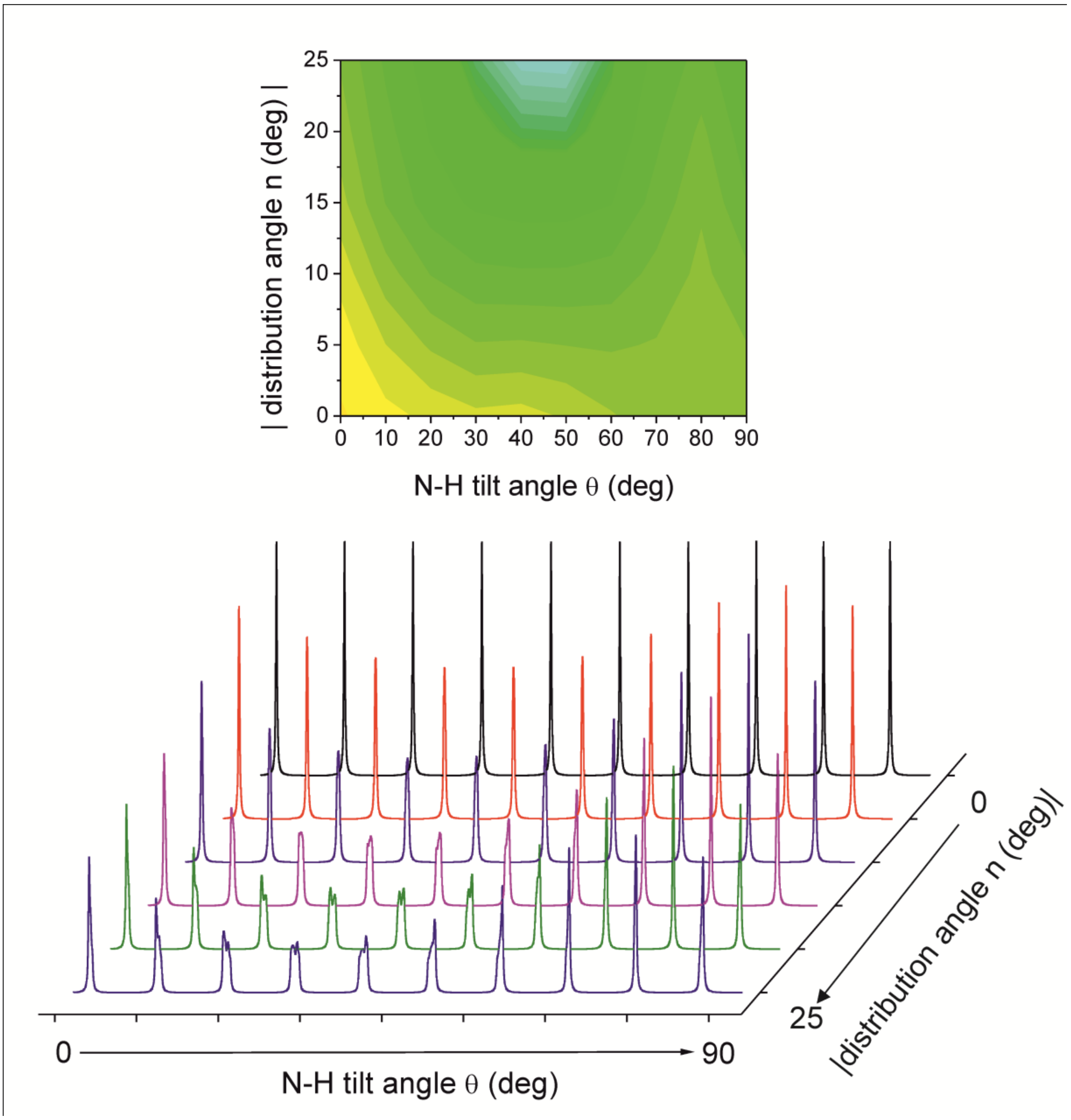

Figure S7. Simulated ^{15}N lineshapes calculated for the 60 models with the A6K β-strand axis perpendicular to the nanotube axis and the nanotube axis parallel to the magnetic field (model set *PerpPara*). The inset shows the chi-square map obtained by comparison of the experimental ^{15}N spectrum with each of the simulated line shapes.

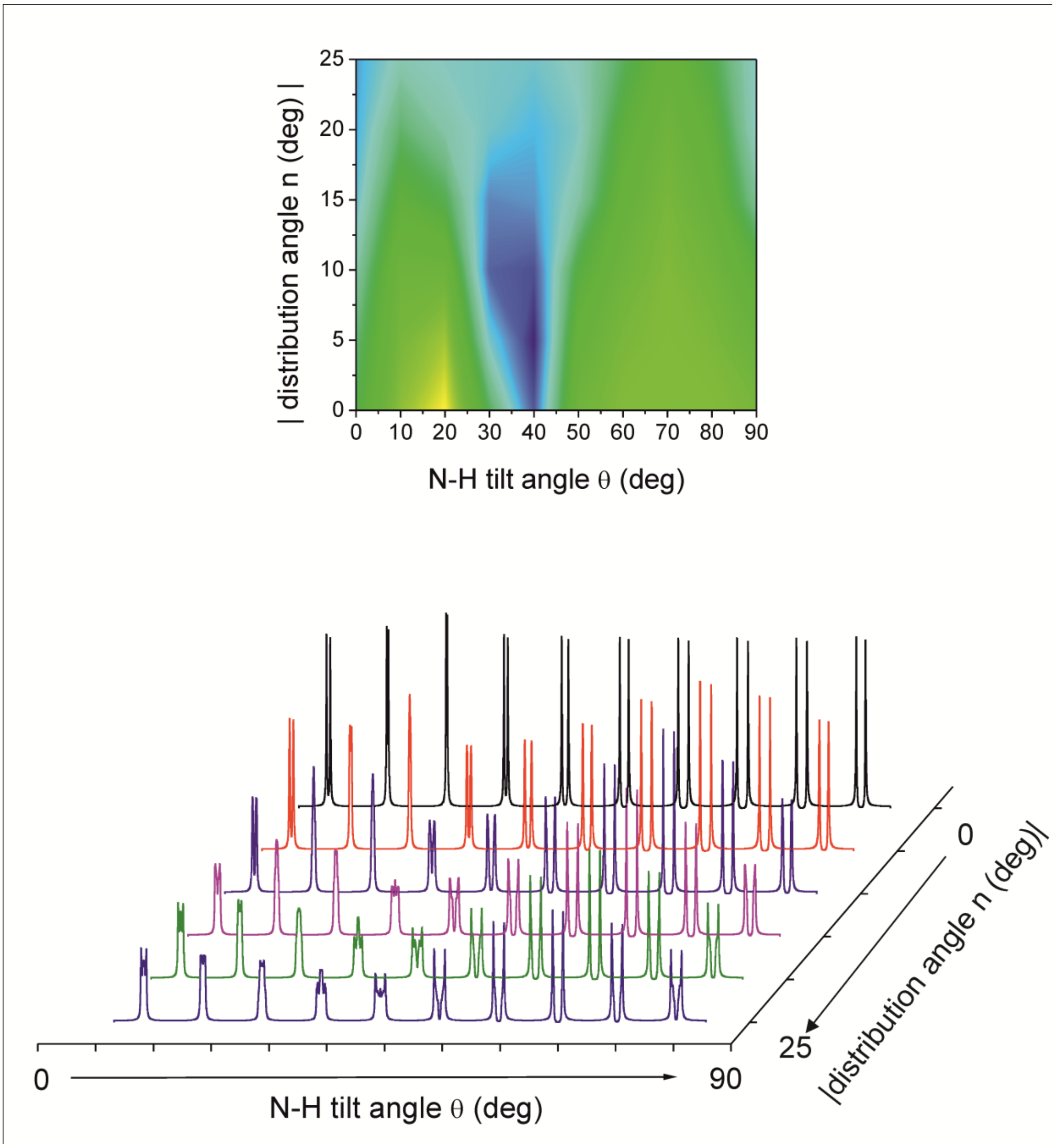

Figure S8. Simulated ^{2}H lineshapes calculated for the 60 models with the A6K β-strand axis perpendicular to the nanotube axis and the nanotube axis parallel to the magnetic field (model set *PerpPara*). The inset shows the chi-square map obtained by comparison of the experimental ^{2}H spectrum with each of the simulated line shapes.

References

[1] Bechinger et al., Biochim. Biophys. Acta 1666 (2004) 190-204.

[2] Griffiths et al., *J. Am. Chem. Soc.*, 1995, **117**, 3539-3546.

[3] J. Madine et al., *J. Am. Chem. Soc.*, 2008, **130**, 14990-15001
